# Challenges to Laboratory Monitoring of Direct Oral Anticoagulants

**DOI:** 10.1177/10760296241241524

**Published:** 2024-04-22

**Authors:** Jesse Qiao, Minh-Ha Tran

**Affiliations:** 1 Irvine Department of Pathology and Laboratory Medicine, University of California, Orange, CA, USA

**Keywords:** direct oral anticoagulants, direct thrombin inhibitor, anticoagulants, coagulation, bleeding reversal, rivaroxaban

## Abstract

Direct oral anticoagulants (DOACs) exert anticoagulation effect by directly inhibiting Factor Xa (rivaroxaban, apixaban, and edoxaban) or thrombin (dabigatran). Though DOACs are characterized by fixed-dose prescribing and generally do not require routine laboratory drug-level monitoring (DLM), circumstances may arise where the DLM may aid in clinical decision-making, including DOAC dose adjustment, anticoagulant class change, or decisions to withhold or administer reversal agents. We review the current literature that describes high-risk patient groups in which DLM may be beneficial for improved patient anticoagulation management and stewardship. The review also summarizes the limitations of conventional coagulation testing and discuss the emerging utility of quantitative methods for routine and rapid emergent evaluation of DOAC drug levels—in particular, the Anti-Xa activity to detect Factor Xa Inhibitors (rivaroxaban, apixaban, and edoxaban). Both technical and regulatory barriers to widespread DLM implementation are limiting factors to further clinical research that must be overcome, in order to propose universal DOAC DLM strategies and provide clinical-laboratory correlation to formally classify high-risk patient groups.

## Introduction

The use of vitamin K antagonists (VKAs) for anticoagulation therapy is challenged by numerous drug and dietary interactions, narrow therapeutic ranges, and routine laboratory monitoring with frequent dose adjustments based on the international normalized ratio (INR).^1^ During the mid-2010s, direct oral anticoagulants (DOACs) became the first-line therapy for several commonly encountered thrombotic indications, including the prevention and treatment of venous thromboembolism (VTE) and stroke prevention in patients with nonvalvular atrial fibrillation (AF). To date, several DOACs have entered clinical use. Direct Factor Xa Inhibitors (DXaI) include rivaroxaban, apixaban, and edoxaban, while dabigatran is the oral direct thrombin inhibitor (DTI). Primary advantages of DOACs, compared to VKAs, include fixed dosing, rapid onsets of action, and fewer reported drug interactions when they entered into routine clinical practice.^2,3^

Although DOACs are generally thought to provide at least similar efficacy and safety profiles to VKAs,^4,5^ there remain unaddressed concerns pertaining to pharmacokinetics and patient risk factors that lead to unprecedented or unexplained bleeding and thrombotic events. Between 2017 and 2019, anticoagulation agents, including DOACs, have surpassed antiplatelets, antibiotics, opioids, and diabetic agents as the number one medication class leading to emergency department (ED) visits for adverse drug events in the United States, accounting for over 20% of total ED visits.^6^

In this article, we aim to review clinical scenarios that highlight potential benefit and utility of DOAC drug-level monitoring (DLM) which would support efforts to increase and improve anticoagulation stewardship among health care systems. We also summarize the current strategies and limitations pertaining to testing, concluding with strategies to standardize.

## Challenge #1: How Can We Classify “High Risk” Patients That May Benefit From DLM?

### Caveats of the Initial DOAC Clinical Trials

Clinical trials of DOACs were extensively evaluated their safety and efficacy in different patient groups, each containing a group of “high risk” subjects. Table 1 lists an overview of each DOAC's clinical trial and discusses exclusion criteria. It is noted that these 4 DOAC clinical trials only compared patients treated for VTE and stroke prevention in nonvalvular AF. The RE-LY Trial comparing dabigatran with warfarin in patients with AF and included a significant elderly population (considered high risk due to increased bleeding and thrombotic risks) and patients with prior stroke and transient ischemic attacks.^7^ The ROCKET AF Trial comparing rivaroxaban with warfarin in patients with nonvalvular AF; high-risk factors were defined by stroke, VTE, or having at least 2 risk factors for stroke.^8^ Although the ROCKET AF Trial included patients with moderate renal impairment (creatinine clearance of 30-49 mL/min), a major limitation of both RE-LY and ROCKET AF Trials is the exclusion of patients with severe renal impairment (creatinine clearance of <25 mL/min).

**Table 1.** Study Overview, Regimen Compared, and Exclusion Criteria Pertaining to DOAC Clinical Trials.

Clinical study^a^	Study Demographics^b^	Regimens Compared	Noncardiovascular, Cerebrovascular, or Bleeding Exclusion Criteria^c^
Dabigatran (Connolly et al) RE-LY Trial	Total participants: 18 113 Age (years): 71 (63-80) Weight (kg): 83 (63-102) (Refer to Table 1 of study)	Dabigatran 110 mg Dabigatran 150 mg Warfarin dose adjusted	Concurrent Severe renal impairment (CrCl <30 mL/min) Anemia <10 g/dL Thrombocytopenia <100 k/uL Active liver disease Pregnancy Recent malignancy or radiation therapy within 6 months and not expected to survive 3 years For full list, refer to Supplementary Appendix, Table 2
Rivaroxaban (Patel et al) ROCKET AF Trial	Total participants: 14 264 Age (years): 73 (65-78) BMI: 28.2 (25.1-32.1) (Refer to Table 1 of study)	Rivaroxaban 20 mg (15 mg in CrCl 30-49 mL/min) Warfarin dose adjusted	Medications with cytochrome P450 3A4 inhibition Anemia <10 g/dL Pregnancy or breastfeeding Contraindications to warfarin Known HIV Severe renal impairment (CrCl <30 mL/min) Known significant liver disease or ALT >3× normal For full list, refer to Supplementary Appendix, Exclusion Criteria
Apixaban (Granger et al) ARISTOTLE Trial	Total participants: 18 201 Age (years): 70 (63-76) Weight (g): 82 (70-96) (Refer to Table 1 of study)	Apixaban 5 mg (2.5 mg if 2 of 3 criteria are met: age 80+ years, weight <60 kg, and serum creatinine >1.5 mg/dL) Warfarin dose adjusted	Severe renal insufficiency (creatinine >2.5 mg/dL or CrCl <25 mL/min) The ARISTOTLE consort diagram excluded 2797 patients who “did not meet the inclusion criteria” which included: atrial fibrillation or flutter at the time of enrollment, or documented by ECG twice in the past 12 months, AND at least one risk factor for stroke (refer to “Study Population” in main body of manuscript and Supplementary Appendix for further details).
Edoxaban (Guigliano et al) ENGAGE Trial	Total participants: Age (years): 72 (64-78) Weight/BMI: not examined (Refer to Table 1 of study)	Edoxaban 60 mg (30 mg with any one of following: CrCr 30-50 mL/min, weight 60 kg, use of verapamil or quinidine) Warfarin dose adjusted	Severe renal insufficiency (CrCl <30 mL/min) The ENGAGE Study Consort Diagram excluded 2170 patients who “did not meet the inclusion/exclusion criteria” which included patients >21 years of age with atrial fibrillation documented by ECG within 12 months, score of 2 or higher on CHADS2 risk assessment, and anticoagulation therapy planned for the duration of the trial (refer to “Study Population” in main body of manuscript and Supplementary Appendix for further details).

Abbreviations: BMI, body mass index; CrCl, creatinine clearance; ECG, electrocardiogram; CHADS2, Congestive heart failure, Hypertension, Age ≥75 years, Diabetes mellitus, prior Stroke or TIA or thromboembolism risk assessment profile.

^a^ These 4 DOAC clinical trials pertained to the specific approved use for the treatment of venous thromboembolism, and stroke prevention for nonvalvular atrial fibrillation.

^b^ Reported values are reported as medians; Interquartile ranges are expressed (parentheses).

^c^ In addition to the exclusion criteria listed on this table, each study also has cardiovascular cerebrovascular exclusion criteria, including but not limited to: recent or severe stroke, acute coronary syndromes, use of therapeutic doses of aspirin and/or dual antiplatelet agents, high bleeding risk, and other indications for anticoagulation therapy (including treatment of recent thromboembolism). Please refer to each separate clinical trial for full inclusion and exclusion details.

The ARISTOTLE Trial that compared apixaban with warfarin had a greater inclusion criteria that allowed for a wider spectrum of high-risk patients, including elderly patients, those with prior stroke or systemic embolism, and patients with severe renal impairment (creatinine clearance of <25 mL/min).^9^ The ENGAGE AF-TIMI 48 Trial compared edoxaban against warfarin for nonvalvular AF; high-risk patients included the elderly, those with cardiovascular disease, and patients with moderate renal impairment (creatinine clearance of 30 mL/min and above).^10^

Application of exclusion criteria during DOAC clinical trials may have also inadvertently underrepresented other high-risk groups, including but not limited to: liver disease, morbid obesity, active cancer, and polypharmacy (in particular, patient medications that induce or inhibit p-glycoprotein and/or CYP3A4.^11^ While DOAC DLM is not typically required, consideration of DLM in specific clinical scenarios may help mitigate adverse thrombotic or hemorrhagic events.^12,13^

### Drug-Level Monitoring in Cancer and Malignancy

The use of DOAC for cancer VTE treatment has been supported by trials such as the Hokusai VTE Cancer,^14^ SELECT-D,^15^ ADAM-VTE,^16^ and CASSINI^17^ Trials, which explored the efficacy and safety of edoxaban and rivaroxaban. Hokusai VTE, SELECT-D, and CASSINI Trials highlight a slight increased bleeding risk, particularly in patients with gastrointestinal cancers, suggesting the justification for DOAC DLM in specific cancer cohort subpopulations. However, the ADAM-VTE trial did not demonstrate significant differences in bleeding compared to the dalteparin control group.^16^

Compared to LMWH, a recent review of 4 randomized control studies of 2894 patients highlight similar safety and efficacy of DOACs for treating cancer-associated VTE.^18^ Individual risk factors and cost–benefit analyses in low-resource settings should be considered when selecting cancer patients for DOAC therapy to undergo DLM, including the specific type of cancer, the presence of hepatorenal disease, and long-term goals of therapy.^19,20^ In summary, the justification for DOAC DLM of cancer patients undergoing thromboprophylaxis based on the diagnosis appears multifactorial, based on the type of malignancy and concurrent morbidities that predispose risk.

### Drug-Level Monitoring With Hepatic and Renal Impairment

Recent studies highlight the challenges in managing anticoagulation therapy in patients with hepatic and renal impairment, where fixed dosing may not be adequate or safe due to suspected altered pharmacokinetics of the CYP3A4 (hepatic) and P-glycoprotein (renal) pathways, respectively.^21^ A case report described persistent rivaroxaban effect due to impaired renal clearance and medication interactions, attributing the delayed rivaroxaban clearance due to competing patient medications that are also cleared renally.^22^ Studies that examine inpatient DOACs dosing with renal impairment further address the need for careful dosing and monitoring, given the potential for altered renal clearance that impacts efficacy.^21,23^

Direct oral anticoagulant–specific pharmacokinetics with hepatic impairment in the *absence* of renal impairment are not yet well studied. Although DOAC use is generally discouraged with liver failure, the use of DOACs to treat portal vein thrombosis in cirrhotic patients has been described in a recent systematic review,^24^ it is worth mentioning that subsets of patients with hepatic dysfunction may also have concurrent renal dysfunction. Like patients with renal impairment, concerns pertaining to the safety and efficacy of DOACs in cancer patients with hepatic impairment have also been reported.^25^ These recent studies suggest a cautious approach to DOAC therapy in these populations, given the interplay of cancer, anticoagulation, and hepatorenal dysfunction.^21,25^ In summary, it is generally reasonable to incorporate DOAC DLM for patients with hepatic and/or renal disease.

### Drug-Level Monitoring in Perioperative Settings

Theoretically speaking, DOAC perioperative DLM has the potential to advise clinicians on informed decisions regarding the timing of surgery, managing anticoagulation (including the need for reversal for urgent procedures), implementing aggressive measures to reduce blood loss, and minimizing additional perioperative risks. A case study and literature review emphasized that plasma levels of apixaban may be altered in patients undergoing major gastrointestinal surgery, advocating for DOAC DLM in such patients.^26^ Perioperative DOAC DLM is supported due to concerns pertaining to DOAC pharmacodynamics and pharmacokinetics that impact efficacy and safety in the setting orthopedic surgery.^27^ In a multicenter cohort study of 1065 patients, 232 patients had preoperative DOAC DLM but reported worsened outcomes compared to the patients without preoperative DLM.^28^ Major limitations of this retrospective cohort study include selection bias and lack of randomization; patients who received the most DOAC DLM included high-risk bleeding surgical procedures, including spinal, orthopedic, and neurosurgical procedures. Additional robust randomized clinical trial data may be necessary to draw general conclusions pertaining to perioperative DOAC DLM.

### Drug-Level Monitoring in the Detection of Drug–Drug Interactions and Medication Compliance

Patients who are prescribed therapeutic DOAC doses may infrequently present with recurrent thrombotic or bleeding complications due to medication interactions or compliance. An observational study of 48 442 patients comparing overall and gastrointestinal bleeding of various severities, prescribed rivaroxaban, apixaban, or dabigatran for nonvalvular AF and normal kidney function noted an increased incidence of adverse bleeding events in patients, when the use of DOACs is combined with verapamil or diltiazem use.^29^ A systematic review demonstrated a potential increase in thrombotic risk with the use of antiepileptics, through citing the need for well-designed clinical studies to confirm.^30^

Direct oral anticoagulant DLM has potential to detect known or novel drug interactions that may impact the safety and efficacy of DOAC therapy. A recently published DOAC drug interaction tool^31^ highlights detailed pharmacokinetics and serves as a comprehensive resource for clinicians to navigate the complex landscape of potential medication interactions and their implications for patient care. The highest risk medication groups with interactions include antiepileptic agents, azole antifungal agents, select antibacterial agents, and select antiarrhythmic agents, typically by strong induction or inhibition of CYP3A4 and/or p-gp.^31^

When implemented into system-wide practices, DOAC DLM can positively influence drug compliance and patient anticoagulation management. A quality improvement project involving 318 patients with the general practice surgery clinic showed improvements in DOAC DLM rates, better DOAC clinical follow-up care and counseling, and more appropriate use of laboratory tests.^32^ However, this report did not specify the test methodology used for DOAC DLM.

### Considering Age, Gender, and BMI Extremes for DLM Decisions

The justification for DLM of DOACs for patients with specific considerations such as extreme age (which may be associated with concurrent hepatic and renal comorbidities) and weight extremes where DOAC pharmacokinetics can be significantly altered.^21,33^ A recent pilot study of 38 patients with abnormally low or high body weights show increased or decreased plasma levels due to volume of distribution, respectively.^34^ This study had a limited sample size; the authors attributed the strongest correlation with renal function with plasma levels, a phenomenon that was already identified with prior research. A recent 2021 ISTH SSC subcommittee update that summarizes guidance updates on the use of DOACs in obese patients for VTE treatment and prevention update.^35^ However, the update did not entail specific discussions of DOAC peak, trough, and therapeutic levels in obese patients. Although gender-associated DOAC drug-level variation is not well described, one study described significant gender-specific differences in rivaroxaban peak were between male and female patients, and patients over 60 years of age compared to under 60; no significant differences in apixaban was noted.^36^

### Drug-Level Monitoring With Malabsorption and Gastrointestinal Disease

Direct oral anticoagulant DLM in patients with gastrointestinal disease and malabsorption may be beneficial to ensure adequate dosing for efficacy. Direct oral anticoagulants vary in bioavailability and sites of absorption within the GI tract, which can be significantly affected by surgical resection or bypass of the GI tract.^37,38^ Peak DOAC plasma levels are the most impacted by major GI tract surgeries.^38^ For patients with GI malignancies undergoing resection, DOACs may present additional management challenges, such as the risk of bleeding from luminal primary tumors, and the impact on DOAC absorption and efficacy due to altered anatomy or chemotherapy-induced nausea and vomiting.^39^

### What Can We Conclude From Existing Literature?

Efforts to define and classify “high risk” patients for DOAC monitoring have been based primarily on evidence from single-center cohorts, case series, and retrospective reviews. However, the amount of clinical studies with large sample sizes that specifically compare the use of monitoring with outcomes are limited, with an uncertain level of confidence for the generalization to establish widely adopted guidelines for DLM. We hypothesize that a potential limitation of prior research may be attributed to lack of global laboratory assay availability and standardization to facilitate DLM (in a similar fashion as the INR for warfarin or activated partial thromboplastin time [aPTT] for heparin), which we will discuss in the next section.

## Challenge #2: Can We Standardize Testing for DOAC Monitoring?

**Figure 1.**
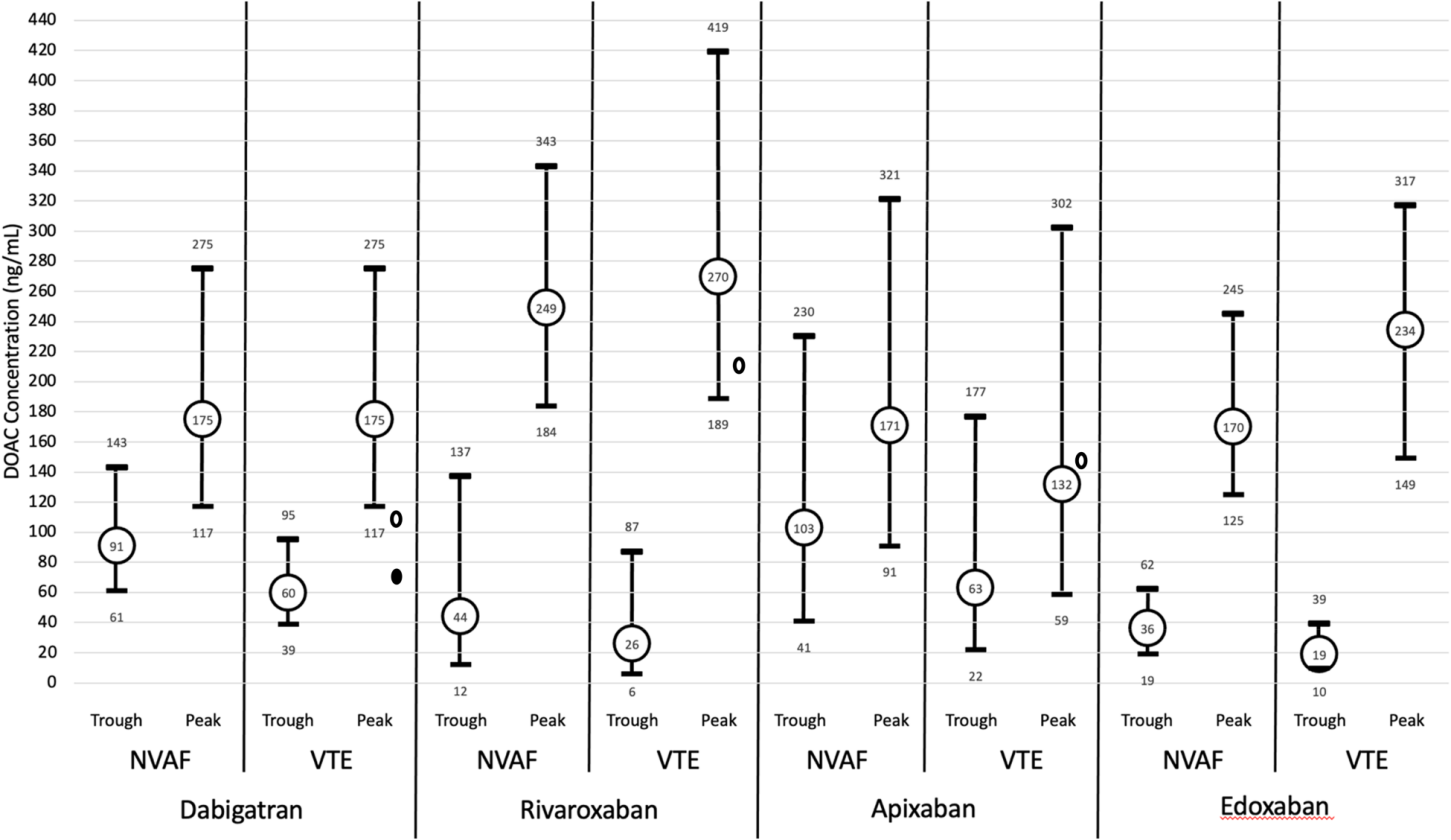
Direct oral anticoagulant (DOAC) peak and trough ranges. Dabigatran = mean and IQR; Rivaroxaban = mean and fifth and 95th percentile; Apixaban = median and fifth and 95th percentile; Edoxaban = median and (1.5 × IQR) for NVAF peak, median, and IQR for remainder—adapted from Gosselin et al.^40^ The small ovals represent median presenting levels for patients in the REVERSE-AD (open oval 110 ng/mL bleeding group; filled oval 73 ng/mL urgent surgery group) and ANNEXA-4 studies (149.7 ng/mL Apixaban; 211.8 ng/mL Rivaroxaban).

Providing interpretation of DOAC levels has been further complicated by the fact the DOAC clinical trials did not formally incorporate DLM.^7–10^ The 2018 International Council for Standardization in Hematology (ICSH[18]) recommendations for DOAC laboratory measurements included expected peak and trough DOAC concentrations for patients being treated for VTE and nonvalvular AF, as depicted in Figure 1 for illustrative purposes.^40^ Our discussion of DOAC laboratory testing methodology below summarizes limitations of screening tests and highlights additional research since the 2018 ICSH recommendations on DOAC testing.

### Limitations of Traditional Coagulation Assays

Routine screening coagulation tests such as the prothrombin time (PT), aPTT, and thrombin time (TT) are neither sensitive nor specific to the presence of DOACs, and do not reflect a linear, dose-dependent response relationships to allow for drug quantification.^41^ The PT is affected less by apixaban, when compared to rivaroxaban.^42^ Edoxaban shows a concentration dependent prolongation of the PT more so than the aPTT.^41^ A normal aPTT excludes therapeutic and supratherapeutic levels but does not exclude drug levels at low-range therapeutic or subtherapeutic levels of dabigatran.^40^ While apixaban shows a dose-dependent prolongation of the aPTT at lower drug levels, this only occurs at plasma levels above 200 ng/mL.^43^ While rivaroxaban shows better sensitivity to the aPTT when compared to apixaban, the aPTT fails to detect rivaroxaban effect at levels less than 50 ng/mL. Edoxaban, however, demonstrates a modest increase in aPTT even after treatment with one dose.^40^ While a normal screening TT excludes presence of dabigatran, an increased TT cannot differentiate between low or high levels of drug, nor differentiate heparin effect.^43^ Finally, PT, aPTT, and TT prolongations may also be attributed to factor deficiencies, inhibitors, and/or global defects of hemostasis.

### Limitations of Viscoelastic Methods

While viscoelastic testing (thromboelastography, rotation thromboelastometry, and sonorrheometry) provides comprehensive whole blot clotting ability beyond what the PT and aPTT may offer, test reagents and results lacks specificity especially in the absence of clinical context. This may be problematic in emergency settings where DOAC ingestion history is not readily available. In addition, normal clotting times (CT or R-times) do not exclude all DOACs. One recent systematic review suggests that the CT for INTEM and EXTEM may be used for the detection of presence of dabigatran, while only CT EXTEM may be used for rivaroxaban in the event of DOAC-associated bleeding.^44^ However, another recent systematic review using 53 studies assessing rivaroxaban and apixaban demonstrated interassay variability,^45^ which may further limit the use of viscoelastic testing alone to differentiate DOAC medication effect.

### Plasma DOAC Levels: “Gold Standard”?

Liquid chromatography-mass spectrometry (LC-MS) remains the gold standard to determine and quantify plasma drug concentrations, using liquid chromatography as a separation technique coupled to a mass spectrometer, which ionizes molecules and then sorts and identifies the ions according to their mass-to-charge (m/z) ratios.^46^ Recent advancements with instrument automation, increased understanding of clinical relevance, and the increasing number of available instrument platforms have increased the application of LC-MS in a variety of clinical settings, including the use to quantify DOAC plasma concentrations.^26,47^ The major limitation of LC-MS remains the labor intensiveness of the assay; testing is typically performed in batches at reference laboratories rather than clinical and hospital laboratories.

### Chromogenic Anti-Xa Activity for Monitoring of Apixaban, Rivaroxaban, and Edoxaban

Although not yet universally standardized across laboratories,^48^ the use of the chromogenic Anti-Xa activity to detect FXaI DOACs has been increasingly reported in recent literature since the 2018 ICSH consensus on DOAC laboratory testing^40^ and was utilized in 4 retrospective cohort studies and one case report discussed in the first section of this review.^22,28,34,36,37^ While the Anti-Xa activity was originally designed to monitor for low-molecular-weight heparin (LMWH) therapy, its use has expanded to unfractionated heparin,^49^ as well as FXaIs. When implemented in a similar fashion as LMWH, these assays have a rapid turnaround time (<30 min) and can achieve lower limits of detection of <20 to <30 ng/mL, sufficient for clinical decision-making to rule out the presence of FXaIs,^50,51^ particularly in the ED setting.

**Figure 2.**
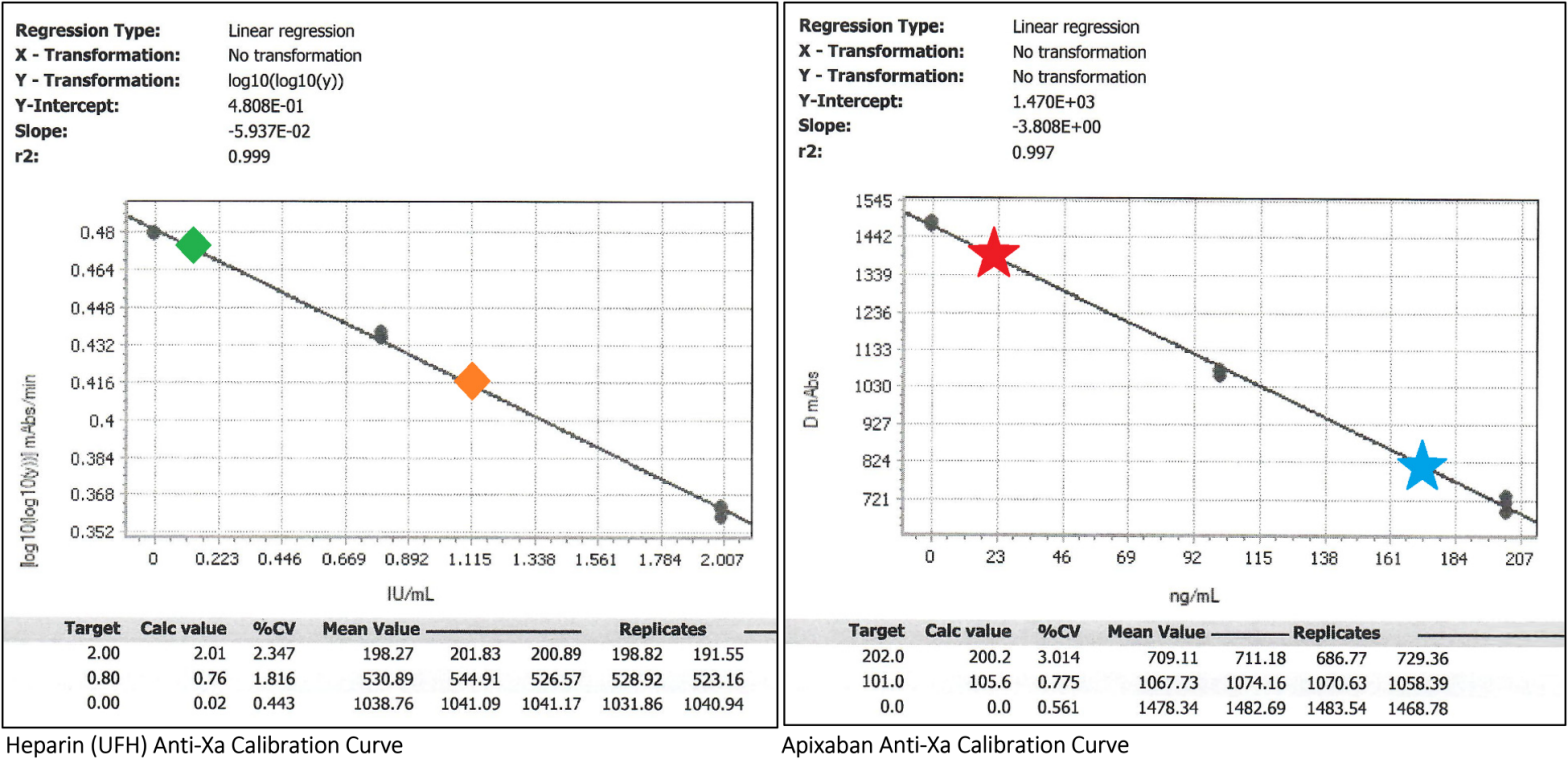
Comparison of unfractionated heparin (UFH) and apixaban anti-Xa calibration curves. *Methodology*: Anti-Xa is performed on a standard coagulation specimen (blue top) tube containing 3.2% sodium citrate and 2.7 mL of patient whole blood, centrifuged to obtain platelet poor plasma for testing. The degree of residual Anti-Xa activity is inversely proportional to the concentration of plasma anticoagulant medication. To perform the Anti-Xa assay (including calibration data points), a fixed concentration of reagent Factor Xa is added to the test plasma. A substrate with chromophore is added and is cleaved by Factor Xa; light emission is detected at 450 nm and measured in absorbance units (mAbs). *Constructing the calibration curve*: The left and right boxes represent calibration curves of Anti-Xa activity using UFH and apixaban drug calibrators, respectively. For each calibration curve, known plasma concentrations of the drug are denoted under the “Target” column (UFH: IU/mL; apixaban: ng/mL) and are plotted on the X-axis. For each known plasma concentration, an Anti-Xa assay is performed based on 3 to 4 replicates (runs) of the same sample (“Replicates” column, with goal Coefficient of Variation <10%—“%CV” column) with subsequent mAbs. The “Mean Value” of mAbs is then plotted on the Y-axis with transformation for heparin (left) and without transformation for apixaban (right). The best-fit line is plotted, with the slope, intercept, and r^2^ as accordingly. *Example interpretations*: The *left green diamond* and *left red star* represent low plasma levels of UFH and apixaban, respectively, as denoted by high Anti-Xa mAbs values. The *right orange diamond* and the *right blue star* represent high plasma levels of UFH and apixaban, respectively, as denoted by low residual Anti-Xa mAbs values due to inhibition of reagent Factor Xa by anticoagulation.

Drug-specific Anti-Xa calibrators have been developed for routine coagulation analyzers and are preferred over screening coagulation assays to monitor FXaIs.^52^ To construct a Anti-Xa drug calibration curve, known concentrations of reagent calibrators containing each specific anticoagulation medication are tested for Anti-Xa and plotted onto a standard curve.^53^ Patient specimens are first measured for residual Anti-Xa activity (which is inversely proportional to the amount of anticoagulation medication present). Then, using the standard curve, the concentration of the medication is determined and resulted.^50,54^ Figure 2 compares a chromogenic Anti-Xa LMWH calibrated curve to an apixaban calibrated curve for use at a routine hospital laboratory facility.

A LMWH-based, calibrated Anti-Xa assay demonstrated significant correlation between Anti-Xa activity compared to LC-MS for rivaroxaban and apixaban (*R*^2^ = 0.947 and 0.959, respectively).^50^ Similarly, an edoxaban-calibrated automated Anti-Xa assay demonstrated good accuracy when correlated with LC-MS.^55^ In addition, the use of a universal, LMWH-based calibrated Anti-Xa assay demonstrated clinical utility for the assessment of rivaroxaban, apixaban, and edoxaban levels for routine DLM.^56^

The main advantages of using calibrated Anti-Xa activity assay for monitoring include high sensitivity, specificity, and good correlation with LC-MS for the quantitative detection of Factor Xa inhibitor DOACs, making it a valuable tool to ensure patient safety, compliance, and optimal dosing.^56–58^ The Anti-Xa is standardizable, reproducible and readily implementable in facilities that already use this methodology to monitor for heparin therapy.^50,59^ Compared to the PT and aPTT, Anti-Xa provides better consistent and reliable anticoagulation monitoring for patients with hepatic impairment,^60^ or patients with isolated prolonged PT or aPTT.

There are several disadvantages of the calibrated Anti-Xa assay for FXaI DLM. Despite efforts at standardization, assay variability across different instrumentation may affect interchangeability of results between laboratory facilities.^61^ In addition, there is a need for drug-specific calibrators, adding complexity to test implementation and clinician familiarity, especially in settings with limited resources.^59,62^ The requirements of additional cost and laboratory expertise may limit widespread implementation and use in all clinical settings on a 24/7 basis.^63^ Best practice incentives to reduce cost and/or educate both physicians and clinical laboratory scientists may help overcome implementation barriers.

With an ideal DLM strategy, peak ranges are typically drawn ∼2 to 4 h after ingestion, whereas trough levels are drawn immediately prior to the next scheduled dose. In clinical practice, however, the precise timing of the last dose may be unknown. In emergency scenarios, the specific DOAC medication may also be unknown. Thus, one caveat of drug-specific calibrated Anti-Xa assays to quantify FXaI is the need to know the specific FXaI ingested (rivaroxaban, apixaban, or edoxaban) in order to select the drug-specific calibration curve for interpretation. Additional studies on the use of universal Anti-Xa assays^56^ may provide additional insight on identification of FXaI in “unknown” clinical settings.

Another challenge to the Anti-Xa assay is limited Food and Drug Administration (FDA) approval and availability of drug-specific calibrators that are universally adoptable across all coagulation analyzers. Nonetheless, when established by institutions as laboratory-developed tests (LDTs), Anti-Xa is helpful to guide administration of reversal agents, during emergency situations or therapy-related bleeding at critical anatomical sites.^64^ To facilitate regulatory review processes, a comparative study of drug-specific FXaI calibrated Anti-Xa compared to heparin Anti-Xa demonstrated excellent correlation between FXaI Anti-Xa with heparin Anti-Xa (rivaroxaban: r^2^ = 0.9755; apixaban: r^2^ = 0.9652) in patients with serious bleeding or requiring interventional procedures. In this study, heparin Anti-Xa of 2.0 IU/mL corresponds to approximately 250 ng/mL of either rivaroxaban or apixaban.^65^

### Dilute TT for Monitoring of Dabigatran

Specifically tailored to dabigatran, the dilute TT (DTT) is a modification of the screening TT that direct measures dabigatran's anticoagulant activity by assessing its ability to inhibit thrombin.^66^ This DTT is useful for DLM of DTIs, especially in patients with renal impairment or requiring emergent surgery.^67^ Because the TT is ultrasensitive to heparin or dabigatran (thus, for qualitative screening only), modification of the TT into the DTT by diluting patient plasma permits a quantitative assessment of dabigatran.^68^

Unlike the PTT, the DTT shows a consistent dose-dependent response to DTIs^69^ and has a linear correlation with LC-MS over a wide range of concentrations for detection of levels below 50 ng/mL, with specificity of 96%^70^ and capabilities to detect dabigatran levels as low as 40 ng/mL.^71^ A recent multicenter study successfully used the DTT to assess dabigatran peak and trough levels.^72^ Two abovementioned retrospective cohorts studying perioperative and extreme body weight, respectively, both utilized the DTT to estimate plasma dabigatran levels.^28,34^

Use of the DTT for dabigatran DLM shares similar advantages and disadvantages to the Anti-Xa.^48^ While automated coagulation analyzers allow for ease of operator use as an on-demand coagulation assay, the major limitations of DTT include the requirement for individual laboratory implementation as LDTs, interlaboratory variation, and limited assay approval by regulatory agencies across different coagulation instrumentation.^48^

An alternative to the DTT are the Ecarin clotting/chromogenic assays (ECAs). Ecarin (derived from the venom of the saw-scaled viper, *Echis carinatus*) cleaves prothrombin into meizothrombin; the activity inhibited by DTIs.^71^ While ECAs can quantify DTIs, the assay is prone to extreme low patient prothrombin and fibrinogen levels, limiting its utility in hypofibrinogenmia or severe global factor deficiencies.^71^ In addition, ECAs are not widely available as they are labor-intensive and require implementation as LDTs. Furthermore, the DTT were shown to be more reliable compared to ECAs.^68^

### Direct Oral Anticoagulant–Specific Point-of-Care Methods

Qualitative point-of-care (POC) urine dipstick technology has been studied, where a color change within 10 min provides information on the presence of absence of DOAC effect.^73^ The DOAC urine dipstick test has the advantage to reliably exclude plasma DXaI levels <14 ng/mL and DTI levels <19 ng/mL^74^ drug. While this method shows good correlation with plasma levels for the presence of drug^75^ and demonstrates a high negative predictive value,^76^ visual interpretation may be impacted by the presence of protein, hemoglobin, and urobilinogen. This method is not preferred when quantitative Anti-Xa and DTT assays become increasingly available and can be readily included in a single blue top tube to run parallel with screening coagulation assays (PT and aPTT). A novel method used a fluorescent probe-tagged prothrombin derivative to act as the macromolecular substrate to measure residual Anti-Xa activity, demonstrating comparable results between whole blood and plasma.^77^ Future research on whole blood DXaI or DTI (dabigatran) assays may provide POC options for bedside DOAC DLM.

### Additional Considerations

Addressing specificity of results in an emergent scenario remains a topic for future research. While multiple studies compare sensitivity and specificity of Anti-Xa and DTT assays *against* screening coagulation tests, will pairing of the Anti-Xa or DTT *in conjunction with* screening coagulation tests (PT, aPTT, or viscoelastic testing) improve diagnostic ability to infer the presence of a specific DOAC medication in an unknown clinical setting? Use of DOAC screening panels may be a potential research opportunity in the development of universal DOAC DLM methods. Table 2 provides a summary of DOAC DLM testing, including test systems, assays, strengths, and limitations of each method.

**Table 2.** Summary of Laboratory Testing Methods for DOAC Evaluation.

Test Category	Examples	What is Measured	Sample Type	Advantages	Disadvantages
Screening Conventional Coagulation Tests	PT PTT	Clotting time involving the extrinsic (PT), intrinsic (PTT), and common pathways (both)	Plasma^a^	Routinely available 24/7 in most hospital laboratories May be useful to predict high DOAC plasma concentrations	Inconsistent prolongation by DOACs with routine doses Poor and/or nonlinear correlation with DOAC plasma drug levels Nonspecific prolongations in other diseases (eg, liver failure, factor deficiencies, inhibitors) affect specificity Lack of standardized results due to varying reagent sensitivity and methodology (eg, mechanical vs optical clot detection)
Point-of-care Tests	TEG ROTEM	Clotting time, clot strength, and clot stability, by viscoelastic analysis of clot shear modulus properties	Whole Blood^a^	Provides comprehensive data (compared to the PT/PTT) on fibrin and platelet contribution, in addition to clotting time Typically, available 24/7 at facilities that treat mass hemorrhage such as trauma surgery	Nonspecific prolongation of clotting times by other disease conditions (trauma-related coagulopathies, etc) No DOAC-specific reagents or activators available Parameters on clot amplification may not add diagnostic value
	Urine Dipstick	Change in reagent strip appearance that indicates qualitative DOAC presence	Urine	Rapid detection (approximately 10 min) and exclusion of DOAC Good correlation with very low to absent plasma drug levels	Cannot quantify DOAC plasma levels, when detected Interferences by urine protein, hemoglobin, and urobilinogen
Quantitative Tests (use of calibration curves required)	Anti-Xa	Optical signal detection (at 405 nm) of residual chromophore release, with the inhibition of reagent Factor Xa by FXaIs (when present)	Plasma^a^	Method can be made routinely available on coagulation analyzers Good linear correlation with plasma drug levels Results can provide decision to administer reversal and provide feedback on reversal progress	Varying sensitivity to different FXaIs UFH and LMWH also affects Anti-Xa Limited differentiating potential in “unknown” clinical scenarios^b^ Despite recent increases in clinical use, not available 24/7 across all clinical laboratories Difficulty with standardization
	DTT	Clotting time using lower dilutions of thrombin (when not inhibited by dabigatran)	Plasma^a^	Method can be made routinely available on coagulation analyzers Good linear correlation with plasma drug levels Results can provide decision to administer reversal and provide feedback on reversal progress	Despite recent increases in clinical use, not available 24/7 across all clinical laboratories Potential interferences by heparin and very high fibrinogen levels Limited differentiating potential in “unknown” clinical scenarios^b^ Difficulty with standardization
	ECA	Measurement of Ecarin activity conversion of prothrombin to meizothrombin) and degree of inhibition by dabigatran	Plasma^a^	Most specific to dabigatran Insensitive to warfarin and heparin Good linear correlation with plasma drug levels	Requires specialized Ecarin reagent Requires extensive laboratory implementation and expertise Limited differentiating potential in “unknown” clinical scenarios^b^ Difficulty with standardization
	MS	Separation of serum or plasma components using GC or LC, followed by ionization, fragmentation, detection, and quantification of resulting signal intensities	Plasma^a^ Serum^c^	“Gold Standard” for plasma DOAC drug detection, quantification, and for therapeutic drug monitoring, in general High sensitivity and specificity achieved using tandem mass spectrometry (MS/MS) Can differentiate specific medications/substances in “unknown” clinical scenarios^b^	Requires expensive specialized instrumentation and technical expertise to operate and maintain Extensive laboratory-specific development and validation requires, as matrix effect in biological samples may lead to variability in quantification Tedious and labor intensive to set up prior to running samples, thus not available to routine clinical laboratories on an 24/7 basis Not practical for 24/7 screening in hospital emergency departments

Abbreviations: DOAC, direct oral anticoagulants; PTT, (activated) partial thromboplastin time; PT, prothrombin time; TEG, thromboelastography; ROTEM, rotational thromboelastometry; Anti-Xa, chromogenic anti-Xa activity; Factor Xa, activated coagulation factor ten; nm, nanometers; FXaI, factor Xa inhibitors, UFH, unfractionated heparin; LMWH, low-molecular-weight heparin; DTT, dilute thrombin time; ECA, Ecarin clotting/chromogenic assay; GC, gas chromatography; LC, liquid chromatography; MS, mass spectrometry.

^a^ Plasma and whole blood specimens acceptable for coagulation testing typically use 3.2% sodium citrate as the anticoagulant, in a 9:1 whole blood to anticoagulant ratio.

^b^ “Unknown” clinical scenarios can represent (but not limited to) emergency clinical presentations in which patient medication history is not immediately obtainable. In such scenarios, the use of several rapid testing methods listed above (eg, ordering STAT Anti-Xa and DTT tests, when available) may be one approach to evaluate for DOACs.

^c^ Serum is prepared from whole blood specimens, collected in specimen tubes without anticoagulation, and may contain a serum separator.

## Reversal of DOACs and Monitoring

While the ISTH guideline suggests preoperative reversal if >30 ng/mL, the PAUSE study shows that properly timed interruption of DOAC therapy provides effective mitigation of perioperative bleeding risk and that residual levels <50 ng/mL or ≥50 ng/mL alone do not appear to predict surgical bleeding.^78^ Justification for emergent DLM using Anti-Xa or DTT includes the use of a specific reversal agent rather than a “broad supplementation” by prothrombin complex concentrates (PCCs), and to exclude DOAC-related bleeding if below levels of detection.^79^

Idarucizumab is a humanized monoclonal antibody fragment that outcompetes by 350-fold endogenous thrombin for binding of dabigatran and its acyglucuronide metabolites, effectively neutralizing dabigatran anticoagulant effects.^80^ It was approved in 2015 for reversal of anticoagulant effect of dabigatran for patients with life-threatening bleeding, as well as in cases where rapid reversal is needed for urgent and emergent surgical procedures.^80^

Both 4 factor PCCs (4F-PCC) and andexanet alfa have been recently studied and compared for DXaI reversal. While 4F-PCC rapidly supplies vitamin K-dependent procoagulant factors II, VII, IX, and X, Andexanet alfa is a modified Factor Xa decoy molecule engineered to outcompete endogenous Factor Xa for FXaI binding.^81^ While andexanet alfa offers specific, targeted FXaI reversal compared to 4F-PCCs, a recent meta-analysis showed similar outcomes pertaining to anticoagulant reversal, mortality, and thromboembolic rates between the 2 agents,^82^ for patients with intracranial hemorrhage. However, an observational cohort study of 4395 patients showed that andexanet alfa-treated patients with intracranial hemorrhage or gastrointestinal bleeding were associated with 50% lower odds of in-hospital mortality, compared to those who received 4F-PCC for FXaI reversal.^83^

Anti-Xa activity assays provide clinical decision guidance on whether to administer the specific reversal agent of andexanet alfa,^64^ as undetectable Anti-Xa activity excludes presence of FXaIs. Monitoring for FXaI reversal is relatively straightforward with a follow-up Anti-Xa activity assay. The 2021 ANNEXA-4 substudy demonstrated reduction in Anti-Xa activity in FXaI-treated patients with intracranial hemorrhage with andexanet alfa.^84^ The 2023 final study report of andexanet alfa for major bleeding with FXaIs evaluated a single cohort of 479 anticoagulated patients meeting major bleeding criteria (predominantly intracranial or gastrointestinal bleeding). These patients presented with Anti-Xa activity above predefined thresholds (>75 ng/mL for apixaban and rivaroxaban; >40 ng/mL for edoxaban), received andexanet alfa, and achieved a follow-up Anti-Xa activity which was at nadir (range 10.0-24.4 ng/mL).^85^ A follow-up Anti-Xa activity may be helpful to assess reversal efficacy, as one study demonstrated concentration-dependent differential neutralization of FXaI (using andexanet alfa of 50 vs 100 µg/mL) that was also dependent on the specific type of patient FXaI therapy.^86^

Like Anti-Xa, the DTT provides clinical decision guidance on whether to administer the specific reversal agent of idarucizumab.^48^ A recent study on dabigatran reversal in the setting of major GI bleeding demonstrated a reduction in DTT and ECA following reversal with idarucizumab.^87^ Additional studies on the use of DTT or ECA for dabigatran reversal are limited in the literature, perhaps due to the lack of widespread assay availability.

An algorithm has been proposed that utilizes a combination of viscoelastic tests, urine testing, and quantitative Anti-Xa/DTT assays, in conjunction with consideration of major or critical bleeding (Refer to Figure 1 of Heubner et al).^79^

## Perspectives: A “Catch-22” Scenario?

### Barriers to Implementing DOAC DLM

Recent clinical studies and reviews conclude that further research is necessary to draw definitive conclusions pertaining to the selection of patients to monitor for DOAC anticoagulation therapy. To perform additional larger-scale, patient group-specific research, drug-specific Anti-Xa and DTT assays should be readily widely available, standardized, and familiar to the routine coagulation laboratory. However, the lack of widespread availability of drug-specific assays approved for routine clinical use, may be in part, due to the limited clinical data that supports the clinical outcome benefits by monitoring using therapeutic ranges, which circles back to the need for additional clinical research.

Until FDA-approved DOAC DLM assays become widely available, laboratories must continue to implement DLM as LDTs developed and validated by each performing laboratory. A recent proposed rule in 2023 by the FDA^88^ seeks to assume regulatory oversight on the clinical validation and implementation of LDTs. This proposed rule, if taken into effect, may discourage laboratories from implementing LDTs due to additional extensive clinical validation requirements, which in turn, may limit advancements with DOAC DLM clinical research. The reader is also encouraged to read the response from the College of American Pathologists to the proposed regulatory rule.^89^

## Conclusion

Given the current research, it is reasonable to suggest the routine use of DOAC DLM in scenarios that were excluded in clinical trials (Table 1), and when encountering any “high risk” circumstances as outlined in the first section of our review. Off-label uses of DOACs, such as the use in valvular cardiovascular conditions, application of nonstandard doses, use in electrophysiology procedures, and use in heart transplants, may further contribute to additional risk potential associated with DOAC use.^90^ Providers are encouraged to incorporate individualized patient care decisions, for example, considering Child-Pugh scores^91^ in cirrhotic patients (in particular, categories B and C) to assess “high risk” status when considering DOAC therapy.

With the recent phase 2 clinical trials of the use Factor XI inhibitors^92,93^ and tissue factor inhibitors for anticoagulation therapy and thromboprophylaxis,^94^ the list of medications in the “DOAC” will inevitably continue to expand. As the list of available anticoagulation medication grows and eventually become available for clinical use, it is in the best interest of the general patient population to develop and formalize guidelines and testing strategies, to improve anticoagulation stewardship.
